# β2-microglobulin induces depressive- and anxiety-like behaviors in rat

**DOI:** 10.1371/journal.pone.0198027

**Published:** 2018-05-24

**Authors:** Ping Zhang, Dan Zeng, Yi-Li Yi, Yi-Yun Tang, Wei Zou, Xue-Feng Yang, Chun-Yan Wang, Xiao-Qing Tang

**Affiliations:** 1 Department of Neurology, Affiliated Nanhua Hospital, University of South China, Hengyang, Hunan, China; 2 Institute of Neuroscience, Hunan Province Cooperative Innovation Center for Molecular Target New Drug Study, University of South China, Hengyang, Hunan, China; 3 Department of Physiology, Medical College, University of South China, Hengyang, Hunan, China; 4 Department of Gastroenterology, Affiliated Nanhua Hospital, University of South China, Hengyang, Hunan, China; 5 Department of Pathophysiology, Medical College, University of South China, Hengyang, Hunan, China; Shanghai Mental Health Center, CHINA

## Abstract

β_2_-microglobulin (B2M), the light chain of major histocompatibility complex class I (MHC I) molecules, has been found to impair hippocampal neurogenesis. Based on the crucial role of hippocampal neurogenesis disturbance in the process of depression and anxiety, the aim of the present study is to investigate whether B2M leads to depressive- and anxiety-like behaviors. We found that 6 days after intracerebroventricular injection with B2M (0.3 μg), the immobility times of rats in the tail suspension test and the forced swimming test were increased, the swimming and climbing time in the forced swimming test was decreased, and the latency to feed in the novelty-suppressed feeding test was increased, indicating that B2M induces depressive-like behaviors. In addition, in the elevated plus maze test, B2M-treated rats displayed obvious decline in the number of entries into and the proportion of time spent in the open arm, while the number of total arm entries was no change, which indicated that B2M induces anxiety-like behaviors. Our present findings suggest that target B2M might represent a novel approach for treatment of depression and anxiety.

## Introduction

Depression and anxiety are common and serious psychiatric conditions that cause high morbidity, disability and mortality [1, 2]. There is mounting evidence that abnormal adult neurogenesis is closely related to many psychiatric and mental disorders [3–5]. Adult neurogenesis is a complex sequential process that produces new neurons in the brains of adult mammal throughout life span [6]. Adult neurogenesis is impaired in multiple rodent chronic stress models of depression and anxiety [7–11]. Animal studies have shown that decrease in adult neurogenesis could induce depressive-like behavior [12, 13]. In previous studies, adult hippocampal neurogenesis has been definitely impaired in the transgenic animals, which is significantly involved in anxiety-related behaviors [9, 14]. Furthermore, diverse antidepressants boost adult neurogenesis in stressed animals [15–17] and adult neurogenesis is required for antidepressant efficacy [11, 18]. As well, recent studies also indicated that neurogenesis played a pivotal role in effective treatment for neurological and psychological disorders [19, 20]. Simultaneously, it appears likely that adult hippocampal neurogenesis has significant effect on human brain functions [21, 22] and induce various brain disorders[23]. Thus, impairment in neurogenesis is a key pathophysiological mechanism of depression and anxiety.

The most major histocompatibility complex class I (MHC I) molecules are known for their role in the vertebrate adaptive immune response[24]. Functional MHC I molecules are composed of a transmembrane heavy chain, a soluble β_2_-microglobulin (B2M) light chain, and a 9–11 amino acid peptide[25]. B2M plays an important role in the assembly and cell surface expression of functional MHC I molecules[26, 27]. Increased level of soluble B2M has been observed in the cerebral spinal fluid of patients with HIV-associated dementia [28], Parkinson’s diseases [29], and Alzheimer’s disease [30], as well as implicated in cognitive impairments related to chronic hemodialysis [31, 32]. Recently, B2M is deemed to impair hippocampal neurogenesis and synaptic plasticity [33]. Thus, we speculated that B2M may induce depressive- or anxiety-like behaviors. Furthermore, several studies identify that the levels of B2M is increased in the serum or urine of major depressive disorder [34–36]. In addition, B2M also was considered as a housekeeping gene for discriminated asthma with or without depression[37]. Therefore, it is worth to investigating whether B2M leads to depressive- or anxiety-like behaviors.

In the present study, we found that B2M increased the immobility times of in the tail suspension test and the forced swimming test, extended the latency to feed in the novelty-suppressed feeding test, and declined the number of entries into and the proportion of time spent in the open arm in the elevated plus maze test. Our data suggest that B2M play a critical role in the depressive- and anxiety-like behaviors. Regulation of the levels of B2M might reveal a novel therapeutic approach for the treatment of depression and anxiety.

## Materials and methods

### Animals

Adult male Sprague-Dawley (SD) rats (220–260 g, n = 30), provided by the SJA Lab Animal Center of Changsha (Changsha, Hunan, China), were housed individually in a temperature (22 ± 2°C) and humidity (55%±5%) constant room. Rats were under a 12:12 h light: dark cycle (lights on at 07:00 a.m.) and given free access to standard food and water. The rats were habituated to experimenter and housing conditions for at least 2 weeks before they were tested. In order to avoid information bias, all behavior tests were performed in a blinded manner (observations were removed from analyses). Experiments procedures were carried out according to the National Institute of Health’s Guide for the Care and Use of Laboratory Animals and were approved by the Animal Use and Protection Committee of University of South China. All efforts were made to minimize the amount of animals use as well as their suffering.

### Lateral ventricle cannulation

Rats were anesthetized by administration of 60 mg/kg intraperitoneal sodium pentobarbital (Sigma, St. Louis, MO, USA) before being mounted in stereotaxic apparatus with an incisor-bar and ear-bars; under aseptic conditions, a guide cannula (Rui wo de, Shengzhen, China) was implanted into the right lateral ventricle of rats (1.0 mm posterior to Bregma, 2.0 mm right of the midline, depth of 4 mm below the surface of the skull). The cannulas (5 mm) were permanently attached to the skull with the help of three stainless steel screws and dental cement. After stereotaxic surgery, rats were treated with penicillin (200000 U, intraperitoneally) for two consecutive days and were observed the behavior and wound healing for 7 recovery days in individual feed cage; In order to certify the cannula placement was correct, we monitored the drinking response after injection of 20 ng AngII (Sigma, St. Louis, MO, USA) into right lateral ventricle. Those consuming more than 5 ml of water in 30 min in response to i.c.v. injection of AngII (a rate of 1 μl/min, 5 min) were confirmed the cannula placement properly and used in the study[38].

### Drugs and treatments

**Fig 1:**
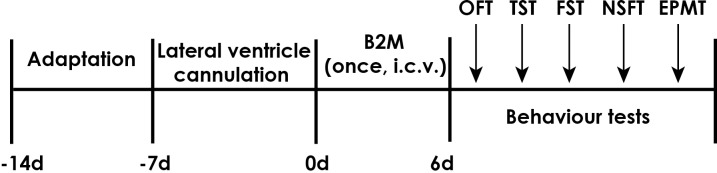
Schematic diagram of the experimental schedule. B2M, β_2_-microglobulin; OFT, open filed test; TST, tail suspension test; FST, forced swimming test; NSFT, novelty suppressed feeding test; EPMT, elevated plus-maze test; i.c.v., intracerebroventricularly.

β_2_-microglobulin (B2M) were obtained from Sigma (Sigma, St. Louis, MO, USA). Male SD rats were randomly divided into two different treatment groups: the control group, the B2M-treated group. B2M were dissolved in phosphate-buffered saline (PBS). B2M or vehicles (PBS) were respectively administered into the lateral ventricle in a volume of 3 μl (0.1 μg/μl, a rate of 0.5 μl/min, single dose) and behavior tests were conducted six days after the injection. The experimental schedule has shown in Fig 1.

### Open field test

An open field test (OFT) was performed to assess the locomotor activity. Locomotor activity in rats was tested in a quiet open field using a locomotion analyzing system (JLBehv-LAR-1; Shanghai Jiliang Software Technology Co. Ltd). The apparatus consisted of a 60 × 60 × 40cm square arena with walls painted black. Briefly, rats were placed in the central of the open-field equipment and allowed to freely explore the area for 5 min. The total distances travelled in the open area was recorded and calculated with a video camera link to computer and regarded as a parameter of motor ability. The apparatus was cleaned between each rat, using 70% ethylalcohol.

### Tail suspension test

The tail suspension test (TST) in rats was performed as described by our previous reports [39, 40]. In brief, the rats were suspended with a hook by approximately 1 cm from the tip of the tail using adhesive tape (distance from the floor was 50 cm). The experimental period was videotaped and the duration of immobility time was observed for 6 min. Immobility was considered as the animal remaining motionless or making only minor, non-escape-related movements. To prevent visual and acoustic contact between the experimental subjects, the animals were individually placed in the TST apparatus.

### Forced swimming test

The forced swimming test (FST) was conducted according to the method described by previously report [41]. The rats were placed individually to swim in a plastic cylinder (height: 40 cm, diameter: 20 cm) filled with 15 cm of 25 ± 1°C water. The total duration of the stress exposure and behavior were recorded in digital video during the FST. The experiment consisted of two sessions. In a pretest session, each animal was forced to swim individually for 15 min in the water-filled plastic cylinder as an adaptive drill. Twenty-four hours after, the animals were again subjected to 5-min test session of FST. Videos were analyzed by individuals blind to the experimental conditions, and the immobility behavior and escape responses (such as climbing and swimming) was quantified during the test period.

### Novelty-suppressed feeding test

The novelty-suppressed feeding test (NSFT) was performed according to previous study [42]. The rats were weighed, and food was deprived for 24 h in their cages (water continued to be provided as usually). Approximately 30 minutes of acclimation period, the rats were transferred to a quiet and dark room, placed in a clean novel cage (1 cm of woodchip bedding covered the floor), which a weighed food pellet was putted in the center. Each subject was placed in a corner of the testing arena, and a chronoscope was immediately started. The latency to first feed, defined as sitting their haunches and biting the pellet with forepaws, was recorded. Immediately, the rats were returned to their home cage with a weighed piece of chow for 30 min, the amount of total food intake was measured.

### Elevated plus maze test

Elevated plus maze test (EPMT) was commonly used to assess anxiety-like behavior as previously described with maze size [43, 44]. In the EPM test for rats, two opposite open arms (50 cm × 10 cm) and two opposite closed arms (50 cm × 10 cm × 40 cm) connected by a central square (10 × 10 cm) make up the apparatus, which are located 50 cm above the floor. The rats were individually placed in the central zone facing one of the open arms, video camera mounted above the maze connected to a computer was used to monitored and scored the explore behavior, during 5-min experimental period. The percentage of open-arm entries (open/total entries × 100) and the proportion of time spent in the open arms (open/total time spent × 100) were calculated for each animal. The more decreased in open arm activity, the more reflected anxiety behavior [45].

## Results

### 1. B2M induces depressive-like behavior in the tail suspension test (TST)

**Fig 2:**
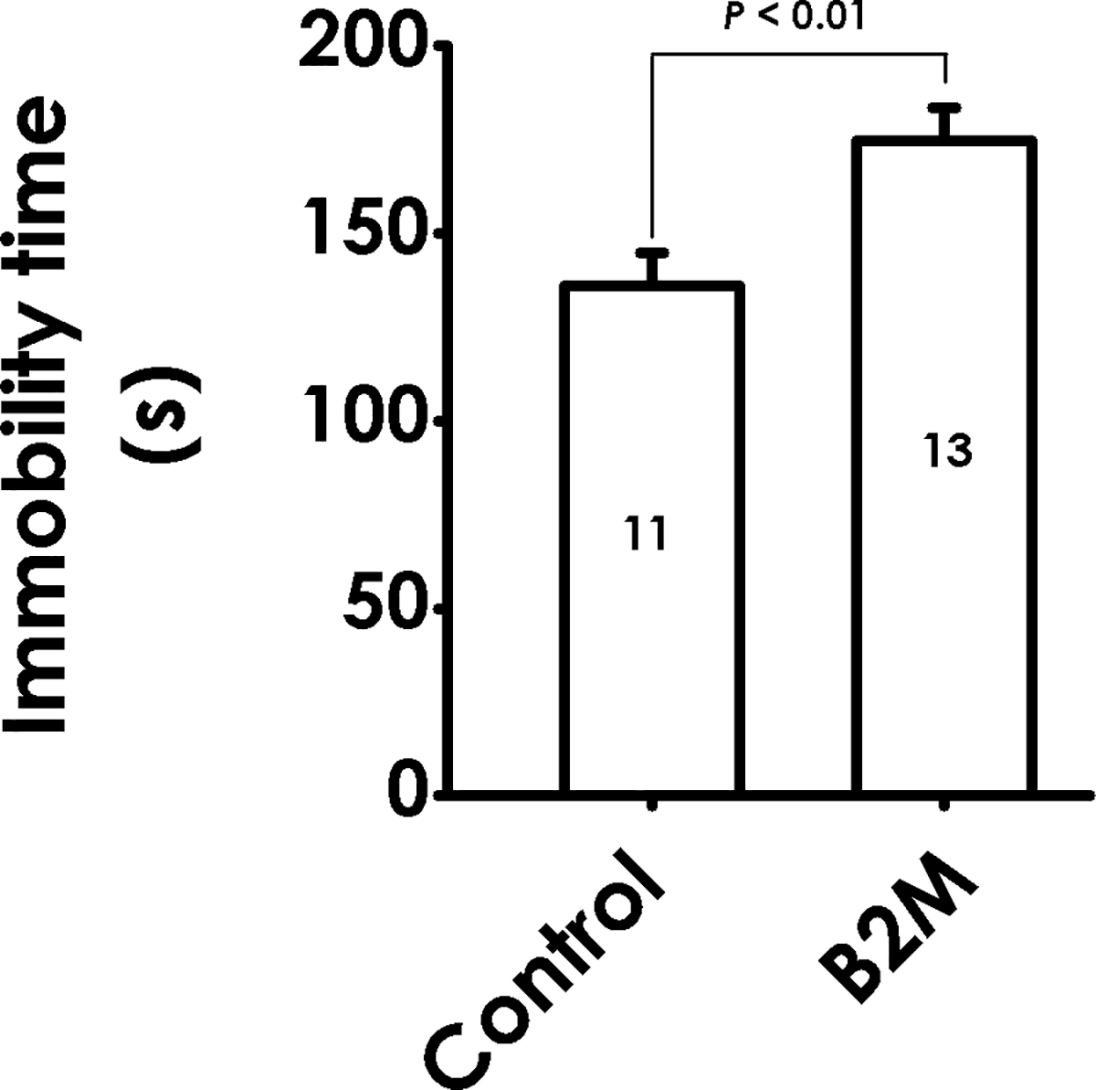
Effect of B2M on the behaviors of rats in the tail suspension test. Six days after intracerebroventricular administration of B2M, the rats were tested by TST for 6 min and the immobility time was record. Values are represented as mean ± SEM, versus control group.

To explore whether B2M induces a depressive-like effect in rats, the TST was performed. B2M significantly extended the immobility time in TST, indicating that B2M triggers depressive-like behavior in rats (Fig 2).

### 2. B2M elicits depressive-like behavior in the forced swimming test (FST)

**Fig 3:**
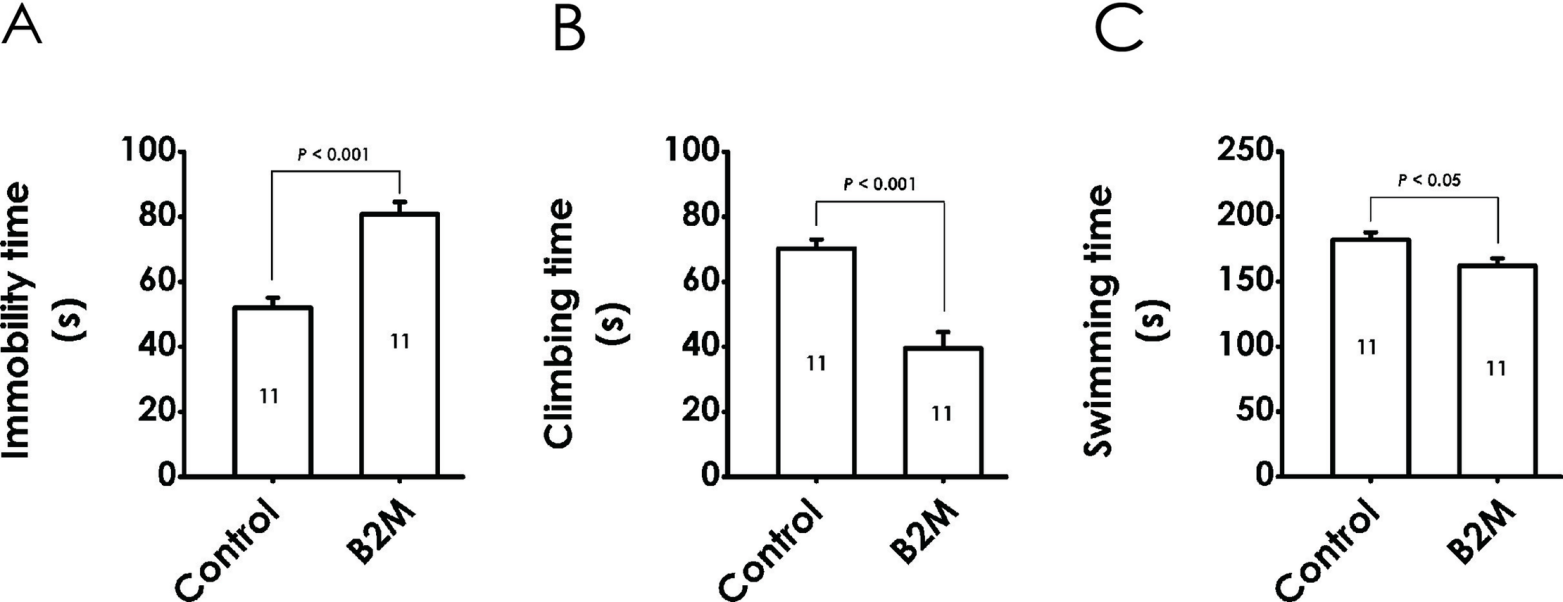
Effect of B2M on the behaviors of rats in the forced swimming test. Six days after intracerebroventricular administration of B2M, the rats were evaluated by the FST for 5 min. The immobility (A), climbing (B), and swimming (C) time during the FST was recorded, respectively. All date are represented as mean ± SEM, versus control group.

The depressive-like effect of B2M was also explored by the FST. As shown in Fig 3, after treatment with B2M, the duration of immobility in the FST was significantly increased (Fig 3A), while the duration of climbing and swimming in the FST were significantly decreased (Fig 3B and 3C). Our data further demonstrated that B2M can elicit depressive-like behavior in rats.

### 3. B2M does not affect the locomotor activity in the open field test (OFT)

**Fig 4:**
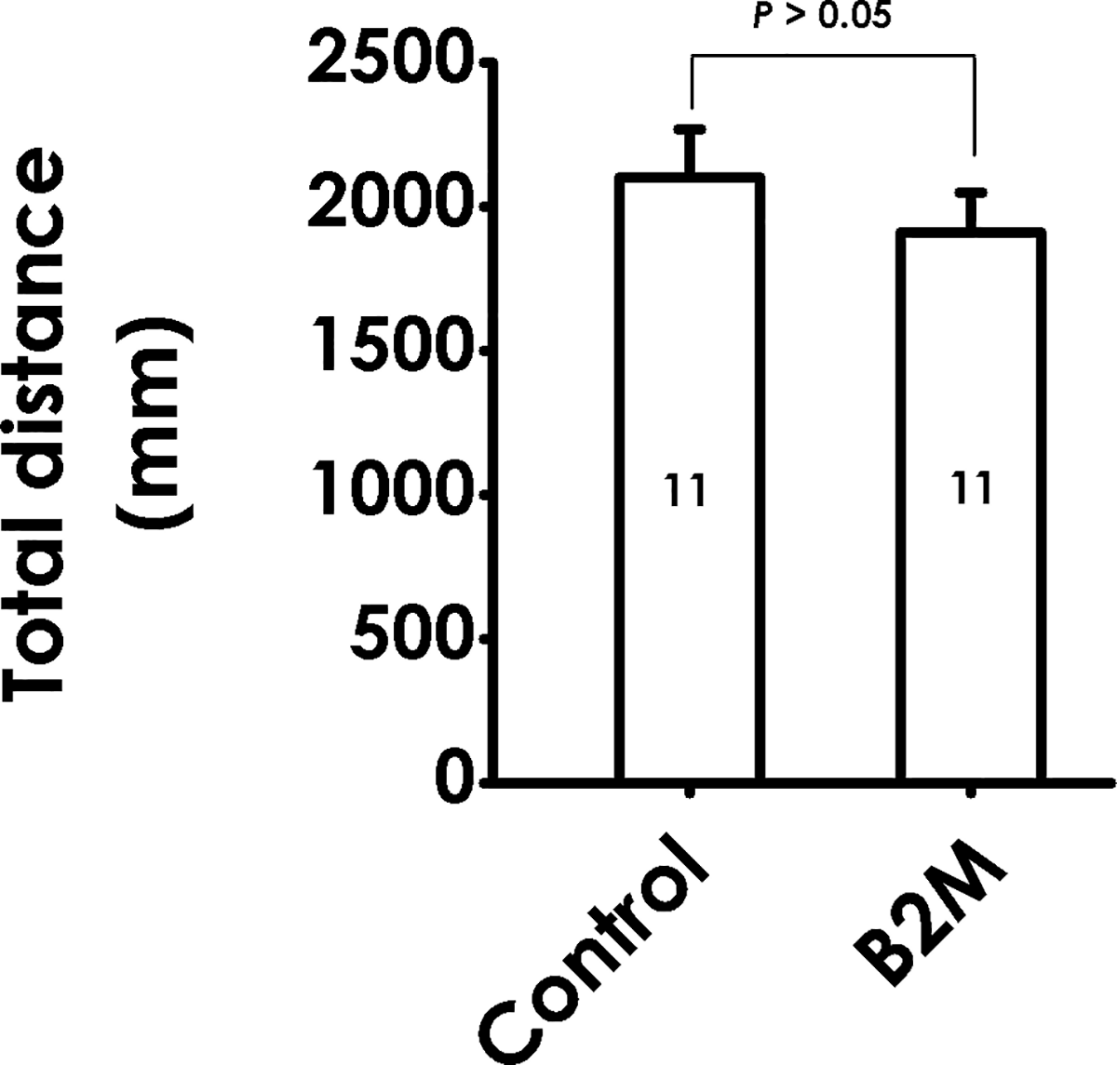
Effect of B2M on the spontaneous activity of rats. Six days after intracerebroventricular administration of B2M, the motor ability of rats were tested by OFT for 5 min and the total distance was recorded. The data are expressed as mean ± SEM.

To eliminate the possibility of B2M-induced muscular contraction involved in behavior activity, we examined the effects of B2M on motor ability in the OFT. No significant effects were observed for total distance travelled between the B2M-treated and the PBS-injected (control) group (Fig 4), indicating that behavior changes in the TST and FST were not responsible for altering the nonspecific motoric.

### 4. B2M exerts depressive-like effect in the novelty-suppressed feeding test (NSFT)

**Fig 5:**
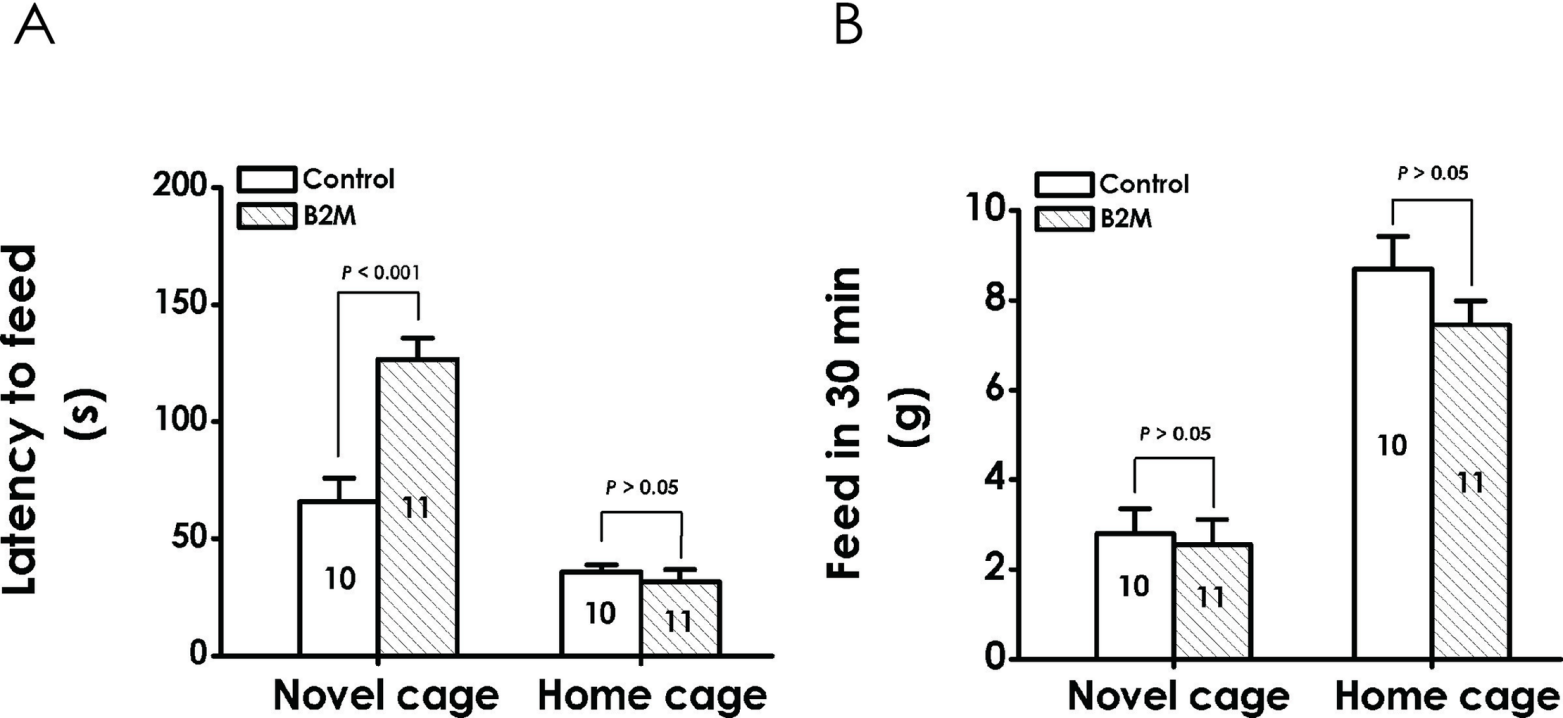
Effect of B2M the behaviors of rats in the novelty-suppressed feeding test. Six days after intracerebroventricular administration of B2M, the rats were assessed by the NSF test. Both latency to feed (A) and feed in 30 min (B) were recorded in the novel and home cage, respectively. Values are expressed as mean ± SEM, versus control group.

To further test whether B2M exerts a depressive-like effect in rats, we also carried out the NSFT. Compared with control group, the time of latency to feed in the B2M-treated group in novel cage was significantly increased, while, there was no significant difference in latency to feed in home cage (Fig 5A). To exclude the possibility that B2M-induced normal appetite and feed intake ability influenced experiment results, we also monitored the food consumption in novel and home cage for 30 min, respectively. As shown in Fig 5B, there was no significant difference on food consumption in 30 min between the B2M-treated group and the PBS-injected group, whether in novel cage or home cage. The aforementioned results confirm that B2M can exert depressive-like behavior in rats.

### 5. B2M produces anxiety-like behavior in the elevated plus maze test (EPMT)

**Fig 6:**
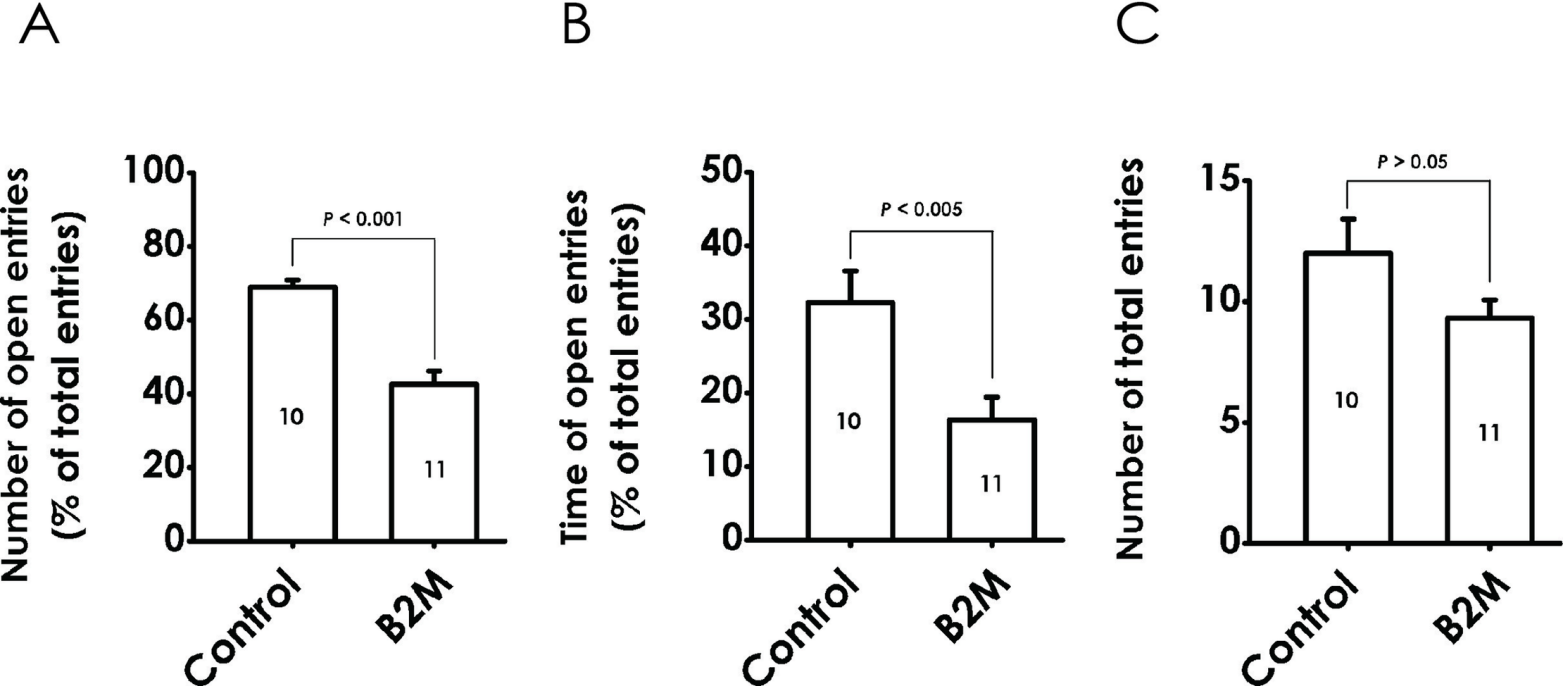
Effect of B2M on the behaviors of rats in the elevated plus maze test. Six days after intracerebroventricular administration of B2M, the rats were tested by the EPM for 5 min. The percentage of open-arm entries (A), the proportion of time spent in the open arms (B) and the number of total arm entries (C) were recorded for each animal. Values are expressed as mean ± SEM, versus control group.

To verify the possibility that B2M produces an anxiety-like effect in rats, we assessed the anxiety level of the rats with the EPMT. B2M-exposed rats showed the anxiety-like behavior defined by decreases in the percentage of open arm entries (Fig 6A) and the time spent in the open arm (Fig 6B), with no significant alter in total arm entries (Fig 6C), suggesting that B2M elicits an anxiety-like effect in rats.

## Discussion

Depression and anxiety are simultaneously experienced [1, 46] and the underlying mechanism remains uncertain, the neurogenic theory might contribute to their pathogenesis [20, 47, 48]. B2M, a pro-aging factor, is known for its toxicity to adult hippocampal neurogenesis [33]. Depression and anxiety disorders tightly associate with adult hippocampal neurogenesis [47, 49]. In the present study, we investigated whether B2M causes depressive- and anxiety-like behaviors. We found that B2M induces depressive-like behaviors in the rats of TST, FST, NSFT and that B2M exerts anxiety-like effect in the rats of EMPT. Our results establish the toxicity of B2M in depression and anxiety disorders.

Accumulating evidence suggests that B2M has closely related to neuropsychiatric disorders. The expression of B2M is significant increase in schizophrenia patients. The mechanism may involve in the impairment of dendritic cell antigen presentation and the imbalance of Th cell differentiation, inducing the cell immune response [50]. In addition, increased level of soluble B2M has been observed in the cerebral spinal fluid of patients with HIV-associated dementia [28], Parkinson’s diseases [29] and Alzheimer’s disease [30]. Therefore, this led us to guess how about the relationship between B2M and depression and anxiety? It has been known that B2M impairs neurogenesis and has related to clinical depression [33, 34]. Furthermore, disturbance of hippocampal neurogenesis is an important pathophysiological mechanism of depression and anxiety. Hence, we investigated whether B2M directly induces depressive- and anxiety-like behaviors in rats.

In this study, administration of B2M results in a conspicuous increase in immobility time in TST of rats, a profound increase in immobility time and a decrease in duration of climbing and swimming in FST, suggesting that B2M produces depressive-like behavior in rats. In addition, treatment with B2M increased the time of latency to feed in novel cage, did not change the food consumption in novel or home cage during the 30 min test session in NSFT, implying that the depressive-like effect of B2M in NSFT was not through affecting normal appetite and feed intake ability. Taken together, our results confirm that B2M can exert depressive-like behaviors in rats.

To eliminate the possibility that B2M induces depressive-like behaviors in rats by influencing muscular contraction, motor ability of rats was evaluated. B2M treatment demonstrated no significant effects on the total distance in open filed test, as compared with control group. The results suggest that the depressive-like effects of B2M in TST, FST, and NSFT are not due to influencing nonspecific motoric.

It is usually identified that depression and anxiety are associated with each other at the same time [1, 46, 48]. On the basis of these researches, it is logical to further hypothesize that B2M may contribute to anxiety-like behaviors in rats. In fact, our results show that administration of B2M for a signal dose obviously decrease both the number of entries into and the proportion of time spent in the open arms of the EPMT, a major confounding factor in this test is the effect of basal locomotor activity, which can be deduced from the number of total entries. As expect, B2M had no effect on the total arm entries. Connectively, our results suggest that B2M dose exert an anxiety-like effect in rats.

Brain-derived neurotrophic factor (BDNF) promotes the regeneration, proliferation and differentiation of hippocampal neurons, and promotes the maturation of newborn neurons [51]. Evidence suggests that, the levels of BDNF were decreased in the models of depression and anxiety, exogenous giving BDNF could ameliorate the depressive- and anxiety-like behaviors, and knockout the expression of BDNF could induce depressive- and anxiety-like behaviors in animals. In addition, BDNF is essential for antidepressant and anxiolytic efficacy[52]. In brief, BDNF has potential role in pathophysiology of many psychiatric diseases or serves as a therapeutic target for treatment of these diseases. It is worthy to speculate whether B2M impairs neurogenesis and induces depressive- and anxiety-like behaviors are relate to inhibiting the expression of BDNF and exogenous giving BDNF can reverse the toxicity of B2M in neuroprotective depression and anxiety disease? Whether other neuroprotective agents can block these courses?

In conclusion, our study provides evidence that B2M induces depression-like behaviors in the TST, FST, and NSF tests of rats. In addition, this study has also demonstrated that B2M produces anxiety-like behaviors in the EPM tests. However, it should be noted that this study has examined only in behaviors tests, the underlying pathophysiological mechanisms that account for this relationship have yet to be elucidated. Findings from this study will provide new direction to uncover the pathogenesis of depression and anxiety diseases. In near future, we will explore specific mechanisms and effective neuroprotectant for blocking the toxicity effects of B2M on depression and anxiety disorders.

## Statistical analyses

All experiments were repeated at least three independent trials. All data are expressed as mean ± standard error of the mean (SEM) and analyses were performed using SPSS 20.0 statistical software (IBM SPSS, Chicago, IL, USA). Statistical significance was determined using one-way analysis of variance (ANOVA) tests followed by Least-significant difference (LSD) test for evaluated within different group, and using Student’s t-test to determined Statistical differences between two sets of groups. Significant main effects (*P* < 0.05) were further analyzed using *post hoc* tests.
